# Impact of Ocean Acidification and Warming on the bioenergetics of developing eggs of Atlantic herring *Clupea harengus*

**DOI:** 10.1093/conphys/coy050

**Published:** 2018-09-18

**Authors:** Elettra Leo, Flemming T Dahlke, Daniela Storch, Hans-O Pörtner, Felix C Mark

**Affiliations:** 1 Alfred Wegener Institute Helmholtz Centre for Polar and Marine Research, Integrative Ecophysiology, Am Handelshafen 12, D-27570 Bremerhaven, Germany; 2 University of Bremen, Fachbereich 2 Biologie/Chemie, NW 2/Leobener Strasse, D-28359 Bremen, Germany

**Keywords:** Atlantic herring, embryonic development, mitochondrial capacity, Ocean Acidification, Ocean Warming, respiration

## Abstract

Atlantic herring (*Clupea harengus*) is a benthic spawner, therefore its eggs are prone to encounter different water conditions during embryonic development, with bottom waters often depleted of oxygen and enriched in CO_2_. Some Atlantic herring spawning grounds are predicted to be highly affected by ongoing Ocean Acidification and Warming with water temperature increasing by up to +3°C and CO_2_ levels reaching ca. 1000 μatm (RCP 8.5). Although many studies investigated the effects of high levels of CO_2_ on the embryonic development of Atlantic herring, little is known about the combination of temperature and ecologically relevant levels of CO_2_. In this study, we investigated the effects of Ocean Acidification and Warming on embryonic metabolic and developmental performance such as mitochondrial function, respiration, hatching success (HS) and growth in Atlantic herring from the Oslo Fjord, one of the spawning grounds predicted to be greatly affected by climate change. Fertilized eggs were incubated under combinations of two *P*CO_2_ conditions (400 μatm and 1100 μatm) and three temperatures (6, 10 and 14°C), which correspond to current and end-of-the-century conditions. We analysed HS, oxygen consumption (MO_2_) and mitochondrial function of embryos as well as larval length at hatch. The capacity of the electron transport system (ETS) increased with temperature, reaching a plateau at 14°C, where the contribution of Complex I to the ETS declined in favour of Complex II. This relative shift was coupled with a dramatic increase in MO_2_ at 14°C. HS was high under ambient spawning conditions (6–10°C), but decreased at 14°C and hatched larvae at this temperature were smaller. Elevated *P*CO_2_ increased larval malformations, indicating sub-lethal effects. These results indicate that energetic limitations due to thermally affected mitochondria and higher energy demand for maintenance occur at the expense of embryonic development and growth.

## Teaser

Ocean Acidification and Warming induce trade-offs in development and growth of Atlantic herring embryos by altering mitochondrial function and increasing the animal’s energy demand.

## Introduction

The atmospheric CO_2_ concentration has increased dramatically since the preindustrial era, from ca. 280 μatm to ca. 410 μatm nowadays causing an increase in average ocean surface temperatures of about 0.83°C and a decrease in surface water pH of 0.1 units (Bopp *et al.*, 2013). If the rate of emissions does not change, the level of atmospheric CO_2_ is expected to rise to ca. 851–1370 μatm by the year 2100 causing an average warming of 3.15°C and a decrease of 0.41 pH units in the ocean surface waters (Henson *et al.*, 2017).

While juvenile and adult fish appear to tolerate CO_2_ levels far beyond average climate change predictions (>2000 μatm, Ishimatsu *et al.*, 2008), early life stages, such as developing embryos and lecithotrophic larvae, appear to be more vulnerable to both, Ocean Acidification and Warming (Baumann *et al.*, 2011; Chambers *et al.*, 2014; Pimentel *et al.*, 2014; Frommel *et al.*, 2016; Stiasny *et al.*, 2016; Dahlke *et al.*, 2017; Sswat *et al.*, 2018). This is probably due to simple modes of respiration (dermal vs. gills) during development and insufficient acid–base regulation before the formation of gills, a situation possibly exacerbated by the higher surface to volume ratio of the early stages compared to adults (Kikkawa *et al.*, 2003; Ishimatsu *et al.*, 2008). Moreover, developing embryos and lecithotrophic larvae are entirely dependent on parental provisioning of resources (yolk) and molecular defence mechanisms (Kamler, 2008) which may become limiting in a changing environment. Exposure to elevated *P*CO_2_ (CO_2_ partial pressure) has been found to adversely affect embryonic development (Tseng *et al.*, 2013; Dahlke *et al.*, 2017), larval growth and survival (Baumann *et al.*, 2011) and tissue/organ health (Frommel *et al.*, 2016) in some fish species. However, in other species, studies have not detected any effect on embryogenesis (Franke and Clemmesen, 2011; Maneja *et al.*, 2015), hatching (Frommel *et al.*, 2012) or growth and development (Munday *et al.*, 2011; Frommel *et al.*, 2012; Hurst *et al.*, 2012, 2013; Bignami *et al.*, 2014). Thus, it is important to understand which are the mechanisms underlying the sensitivity towards Ocean Acidification and Warming in the early life stages to assess which fish species will be affected more by the ongoing climatic changes.

Thermal acclimation and acid–base regulation may increase the metabolic costs of development, causing the reallocation of the yolk-limited resources from development and growth to maintenance (Rombough, 2011; Dahlke *et al.*, 2017). Studies of energy metabolism in developing fish eggs have been concerned mainly with measuring levels of potential energy reserves, metabolites and relevant metabolic enzyme systems (Tocher *et al.*, 1985; Finn *et al.*, 1996; Finn and Fyhn, 2010), and only recently, studies have begun to address how Ocean Acidification and Warming affect the metabolism of fish embryos (Flynn *et al.*, 2015; Pimentel *et al.*, 2016; Dahlke *et al.*, 2017).

In an early study, Boulekbache (1981) described the energetic charge of ATP in the developing embryos of rainbow trout (*Oncorhynchus mykiss*). He found a decrease of ATP/ADP during cleavage (morula–blastula), then a slight increase during gastrulation followed by a plateau. This profile could represent a heavy utilization of pre-existing ATP during the early stage followed by a neosynthesis in parallel to increased embryo cell movement and diversification (Boulekbache, 1981; Tocher *et al.*, 1985). Since the ATP consumption profile seems to be correlated with specific moments of embryonic development and since at these stages ATP production may be limited by the endogenous resources (yolk), ATP production pathways, mitochondrial metabolism in particular, could play a key role in the embryonic sensitivity to ocean acidification and warming.

Atlantic herring (*Clupea harengus*) is a benthic spawner and schooling pelagic fish. It is widely distributed throughout the North Atlantic shelf regions from the East coast of North America to the West coast of Europe and the Baltic Sea Herring populations represent a major resource not only for other fish species, birds and whales (Lynam *et al.* 2015), but also for commercial fisheries, with annual catches of more than 2 million tones (FAO, 2018). Herring population dynamics are known to be sensitive to changes in water temperature: in the last centuries cool periods promoted the increase of herring biomass and the southward expansion of the distribution areas, while warming events exerted the opposite effect (Alheit and Hagen, 1997). Moreover, herring spawn over extended periods with a wide range of spawning locations specific to seasons and populations (Geffen, 2009). Therefore, herring populations spawning in areas predicted to be severely affected by climate changes may be more vulnerable to Ocean Acidification and Warming, especially those spawning during the summer/autumn season. For example, *P*CO_2_ values above 4000 μatm could be reached in the future at important herring spawning grounds in the Baltic Sea such as the Kiel Fjord (Melzner *et al.*, 2013; Frommel *et al.*, 2014) and at higher latitudes including the Skagerrak and North Sea. *P*CO_2_ is predicted to double with an increase in temperature of more than 3°C (Henson *et al.*, 2017; van Vuuren *et al.*, 2011: RPC 8.5). Studies of developing herring embryos from the Baltic Sea found no significant effect of high *P*CO_2_ (4600 μatm) in hatch rate, development, or otolith size (Franke and Clemmesen, 2011). However, no studies have so far addressed the combined effects of increasing temperature and *P*CO_2_ on the embryonic development of this fish.

It is thus important to understand the effects of ocean acidification and warming on the development of herring embryos under the conditions predicted to occur at the spawning grounds by the end of the century. To do so, we incubated developing embryos of Atlantic herring from the Scandinavian coast within a cross-factorial combination of three temperatures (6–10–14°C) and two *P*CO_2_ levels (400–1100 μatm), to mirror present conditions and the conditions projected for the end of the century (RCP. 8.5; van Vuuren *et al.*, 2011; Henson *et al.*, 2017) for the entire developmental period, from fertilization to hatch. We analysed mitochondrial respiration and the oxygen demand of late-stage embryos (50% eye pigmentation) to investigate mitochondrial function in relation to embryonic energy demand, respectively. Furthermore, we observed hatching success (HS), larval deformities and larval length at hatch to identify constraints on performance at the whole-organism level.

## Methodology

This study was conducted at the Sven Lovén Centre for Marine Science, Kristineberg Biological Station (University of Gothenburg, Sweden) between April and May 2013 in accordance with the legislation of the Swedish Board of Agriculture (Permit: 332–2012).

### Experimental animals

Ripe Atlantic herring, *C. harengus*, were caught with gill nets during the spawning season in April 2013 in the inner Oslo Fjord (Norway). Selected fish were caught and killed with a blow on the head and were stored on ice and transported to the Sven Lovén Centre. Gametes of males (*n* = 3) and females (*n* = 3) used for *in vitro* fertilizations were obtained by strip spawning approximately four hours after the fish had been caught.

### Experimental design

A full-factorial design with three temperatures (6, 10 and 14°C) and two *P*CO_2_ (400 μatm and 1100 μatm) was used for fertilization and incubation of herring eggs. Treatment conditions were selected to encompass ambient spawning temperatures (6–10°C and the *P*CO_2_ recorded in the year 2013) as well as water warming and acidification projected for the end of this century according to IPCC’s business-as-usual scenario (RCP 8.5, van Vuuren *et al.*, 2011). Eggs produced by different females were incubated separately and ‘females’ were treated as biological replicates (*n* = 3). Each female was represented by two incubators at each treatment combination (2 × 3 females × 6 treatments = 36 incubators in total). In order to avoid biased survival estimates, only one of both incubators was used to collect samples for measurements of oxygen consumption rates and mitochondrial capacities. The second incubator was used to evaluate HS and larval morphology at hatch. Incubator classification and arrangement within experimental units was done randomly.

### Fertilization protocol

The eggs of each female were stripped onto 12 plates of Polyethylene mesh (500 μm mesh size, 10 cm diameter). To optimize fertilization success and oxygenation during development, care was taken to arrange the eggs in single layer. Two out of 12 egg-plates were fertilized at each of six different temperature × *P*CO_2_ treatment combinations (Table 1) following a wet-fertilization protocol (Geffen, 1999). Individual egg-plates were placed in Petri dishes and incubated for 10 min with a milt-seawater dilution of 1:500 (10 ml, produced with milt aliquots from *n* = 3 males). After being carefully rinsed, the egg-plates were transferred into hatching jars filled with 1 l of filtered (0.2 μm) and UV sterilized seawater (adjusted to the respective treatment combination). The percentage of fertilized eggs on each plate (i.e. fertilization success) was determined by visual inspection under a stereomicroscope after 12 h of incubation. Mean values are shown in Table 1.

**Table 1:** Mean ± SEM fertilization success of Atlantic herring (*Clupea harengus*) eggs fertilized at different levels of temperature and *P*CO_2_. Differences between temperature and *P*CO_2_ treatments were statistically not significant (*F* = 1.9, *P* = 0.192 and *F* = 0.97, *P* = 0.344, respectively)

Temperature (°C)	Fertilization success (%)	
	Control *P*CO_2_	High *P*CO_2_
6	88.8 ± 2.8	79.4 ± 4.3
10	76.0 ± 9.0	72.2 ± 8.6
14	88.9 ± 8.5	85.3 ± 8.0

### Incubation

The incubation set-up is shown in Supplementary Figure S1. Herring eggs adhered to mesh-plates were incubated within transparent, bottom tapered hatching jars (Imhoff sedimentation cones, 1000 ml volume, Supplementary Fig. S1A), which were submerged into 400 l seawater baths thermostatted to different temperatures (6, 10 and 14°C, Supplementary Fig. S1B). Each incubator was sealed with a Styrofoam lid to prevent outgassing of CO_2_. Eggs received dim light with a daily rhythm of 12 h/12 h light/darkness. Every 24 h, 80% of the water volume of each incubator was replaced by filtered (0.2 μm) and UV-sterilized seawater (33 PSU) to avoid oxygen depletion and bacterial or fungal infestation. Herring eggs were not exposed to air during water exchange. Each water bath contained two 60-l reservoir tanks, which were used to pre-adjust exchange-seawater to the corresponding temperature and *P*CO_2_. Water temperatures of the different water baths were recorded automatically every 15 min (±0.1°C, Table 2) by a multi-channel aquarium computer (IKS-Aquastar, IKS Systems, Germany).

**Table 2:** Summary table of the water parameters measured during the incubation of Atlantic herring (*Clupea harengus*) eggs until hatch. Data are presented as mean ± SD

Duration (days)	Nominal *T* (°C)	Measured *T*(°C)	Oxygen (%)		*P*CO_2_ (μatm)		pH_F_	
			Control *P*CO_2_	High *P*CO_2_	Control *P*CO_2_	High *P*CO_2_	Control *P*CO_2_	High *P*CO_2_
27	6	6.15 ± 0.06	94.40 ± 0.71	94.40 ± 0.61	415 ± 10	1101 ± 47	8.15 ± 0.02	7.77 ± 0.02
16	10	10.04 ± 0.06	94.40 ± 0.63	94.40 ± 0.49	408 ± 10	1050 ± 46	8.17 ± 0.02	7.79 ± 0.03
11	14	14.07 ± 0.20	95.00 ± 0.00	95.00 ± 0.00	403 ± 12	1050 ± 29	8.18 ± 0.02	7.78 ± 0.02

Elevated *P*CO_2_ conditions were administered by injection of pure CO_2_ gas into the submerged 60 l reservoir tanks by bubbling through large aeration stones (20 cm length). A multi-channel feedback system (IKS-Aquastar), connected to individual pH-probes (IKS-Aquastar) and solenoid valves were used to adjust *P*CO_2_ values. Pure CO_2_ was infused via perforated silicone tubes until the desired pH/*P*CO_2_ was reached. The *P*CO_2_ of the reservoir tanks was measured *in situ* prior to every second water exchange with an infrared *P*CO_2_ probe (Vaisala GM70, manual temperature compensation, ±10 μatm accuracy; Vaisala, Finland). The probe was equipped with an aspiration pump and sealed with a gas-permeable membrane to measure *P*CO_2_ in air equilibrated with dissolved CO_2_ in the water, as described by Munday *et al.* (2013) and Jutfelt and Hedgarde (2013). Factory calibration was confirmed by measurements of seawater previously bubbled with a technical gas mixture (1010 μatm CO_2_ in air; AGA Sweden). Prior to the daily water exchange, pH-values of the reservoir tanks were measured with a lab-grade pH-electrode to three decimal places (Mettler Toledo InLab Routine Pt 1000 with temperature compensation, Mettler Toledo, Switzerland), which was connected to a WTW 3310 pH-meter. A two-point calibration with NBS-buffers was performed on a daily basis. To convert NBS to the free proton concentration scale for seawater pH (Waters and Millero, 2013), the electrode was recalibrated with Tris-HCl seawater buffers (Dickson *et al.*, 2007), which were acclimated to the corresponding incubation temperature prior to each measurement. Seawater pH-values refer to the free proton concentration scale throughout this manuscript (for summary see Table 2), Individual values for each measured parameter are available in the Open Access library PANGAEA (see ‘Data availability’ section).

### Data collection

#### Whole-embryo oxygen consumption

Oxygen consumption rates (MO_2_) of late-stage embryos (at 50% eye pigmentation) were measured in closed, temperature-controlled respiration chambers (OXY0 41 A, Collotec Meßtechnik GmbH, Germany, Supplementary Fig. S2) following methodologies described by Schiffer *et al.* (2014). All measurements were performed in duplicates (with two respiration chambers) at the same developmental stage (~50% eye pigmentation) and treatment as during incubation. Staging was done by visual inspection during the daily water exchange. Development times until 50% eye pigmentation (and hatching) did not differ between *P*CO_2_ treatments (Supplementary Fig. S3 and Table S1), as was demonstrated for Baltic herring under more extreme *P*CO_2_ conditions (4 600 μatm, Franke and Clemmesen, 2011). The stage at 50% eye pigmentation was selected because it represents a clearly discernible developmental landmark (Hill and Johnston, 1997) at which the embryonic cardiocirculatory system, and thus metabolic capacity, is already well-developed (Hill and Johnston, 1997). For each run, ~20 (±3) eggs were loaded into each of the two respiration chambers. The chambers were previously filled with a volume of ~2 ml sterilized seawater, whereby each chamber was alternately used for different *P*CO_2_ treatments. The eggs were placed on a polyethylene mesh (500 μm mesh size) with a magnetic micro-stirrer (3 mm) underneath to avoid oxygen stratification within the respiration chamber (see Supplementary Fig. S2). The change in oxygen saturation was detected by micro-optodes (fiber-optic microsensor, flat broken tip, diameter: 140 μm, PreSens GmbH, Germany) connected to a Microx TX3 (PreSens GmbH, Germany). Recordings were stopped after 60 min (at 6°C) or as soon as the oxygen saturation declined below 80% air saturation (20–40 min at 10 and 14°C). After each run, the wet mass per egg and the exact water volume of the respiration chamber was determined by weighing on a precision balance (±0.01 mg). Bacterial oxygen consumption (always below 5%) and optode drift (always below 1%) was determined by blank measurements before and after three successive runs with eggs. Given that egg masses did not differ between temperature and *P*CO_2_ treatments (Supplementary Fig. S3), MO_2_ was expressed as (nmol O_2_ egg^−1^ h^−1^) according to the following formula: MO_2_ = DO_2_ * Vol/*N*_Eggs_, where DO_2_ is the decline in oxygen saturation (nmol l^−1^ h^−1^), Vol is the water volume of the respiration (ml) chamber and *N*_Eggs_ is the number of eggs.

#### Mitochondrial function

Mitochondrial function was measured in a cellular suspension of late-stage eggs (at 50% eye pigmentation) as described in Dahlke *et al.* (2017). Briefly, one hundred eggs from *n* = 3 females were gently ground on ice in a glass potter filled with 2-ml ice-cold modified mitochondrial respiration medium MiR05 (0.5 mM EGTA, 3 mM MgCl_2_, 60 mM K-lactobionate, 20 mM taurine, 10 mM KH_2_PO_4_, 20 mM HEPES, 160 mM sucrose, 1 g l^−1^ bovine serum albumin, pH 7.4, 380 mOsmol l^−1^) (Iftikar and Hickey, 2013; Gnaiger *et al.*, 2015). The resulting suspension was collected avoiding the collection of the eggshells and mitochondrial respiration was analysed using Oroboros Oxygraph-2k™ respirometers (Oroboros Instruments, Innsbruck, Austria). The oxygen flux (nmol O_2_ (egg* h)^−1^) was recorded and calculated in real-time using Oroboros DatLab 5.2.1.51 (Oroboros Instruments, Innsbruck, Austria). Measurements were conducted in MiR05 buffer equilibrated to atmospheric *P*CO_2_ and acclimation temperature of the eggs. The *c*O_2_ ranged from atmospheric saturation (ca. 370 nmol ml^−1^) to 150 nmol ml^−1^. A substrate–uncoupler–inhibitor titration (SUIT) protocol was used to investigate the capacities of the single components of the electron transport system (ETS) measured as oxygen consumption attributable to each component (nmol O_2_ (egg* h)^−1^). In detail: ETS capacity was measured by step-wise (1 μM each) titration of carbonyl cyanide *p*-(trifluoromethoxy)phenyl-hydrazone (FCCP) in the presence of Complex I (CI) and Complex II (CII) substrates (10 mM glutamate, 2 mM malate, 10 mM pyruvate and 10 mM succinate). CI, CII and Complex III (CIII) were inhibited by the addition of 0.5 μM rotenone, 5 mM malonate and 2.5 μM antimycin a, respectively. All chemicals were obtained from Sigma-Aldrich (Germany).

#### Hatching success

Once hatching started, free-swimming larvae were collected in the morning, euthanized with an overdose of tricaine methane sulphonate (MS-222) and counted after visual examination for morphological deformities under a stereomicroscope. The incidence of larval deformities was quantified as the percentage of hatchlings that exhibited severe deformations of the yolk sac, cranium or vertebral column. HS, defined as the percentage of non-malformed larvae that hatched from fertilized eggs, was calculated as:

$$\mathrm{HS}=(L_{i}-L_{d})/E_{f}*100$$

where *L*_*i*_ represents the number of hatched larvae, *L*_*d*_ is the number of deformed larvae and *E*_*f*_ is the number of fertilized eggs (shown in Table 1).

#### Larval size at hatch

Subsamples of 10–30 non-malformed larvae of three females at each treatment combination were photographed for subsequent measurements of larval standard length (SL) using Olympus image analysis software (Stream Essentials^©^, ± 1 μm). Only samples obtained from the same daily cohort (during peak-hatch at each temperature treatment) were used for statistical comparison between *P*CO_2_ treatments.

#### Data analysis

All data are presented as mean ± SEM.

Statistical analyses were conducted using R 3.2.0 (R Core Team, 2015) and the level of statistical significance was set at *P* < 0.05 for all the statistical tests.

Normal distribution and homoscedasticy of the data were assessed by Shapiro–Wilk and Bartlett’s tests, respectively.

Multi factorial analyses of variance (two-way ANOVA) including the female parent ID as covariance were used to evaluate whether temperature and *P*CO_2_ and the combination of both factors had an effect on the parameters object of this study. The two-way ANOVA was followed by Tukey’s HSD test for temperature and Student’s *t*-test for CO_2._

In addition, MO_2_ data were analyzed with the female-specific egg mass included in the model as covariate.

The temperature coefficient *Q*_10_ was calculated according to the equation:

$$Q_{10}={\left(R_{1}/R_{2}\right)}^{10/(T2-T1)}$$

*R*: respiration rate

*T*: temperature at which the respiration rate was measured.

## Results

### Mitochondrial function and whole-embryo respiration

**Figure 1:**
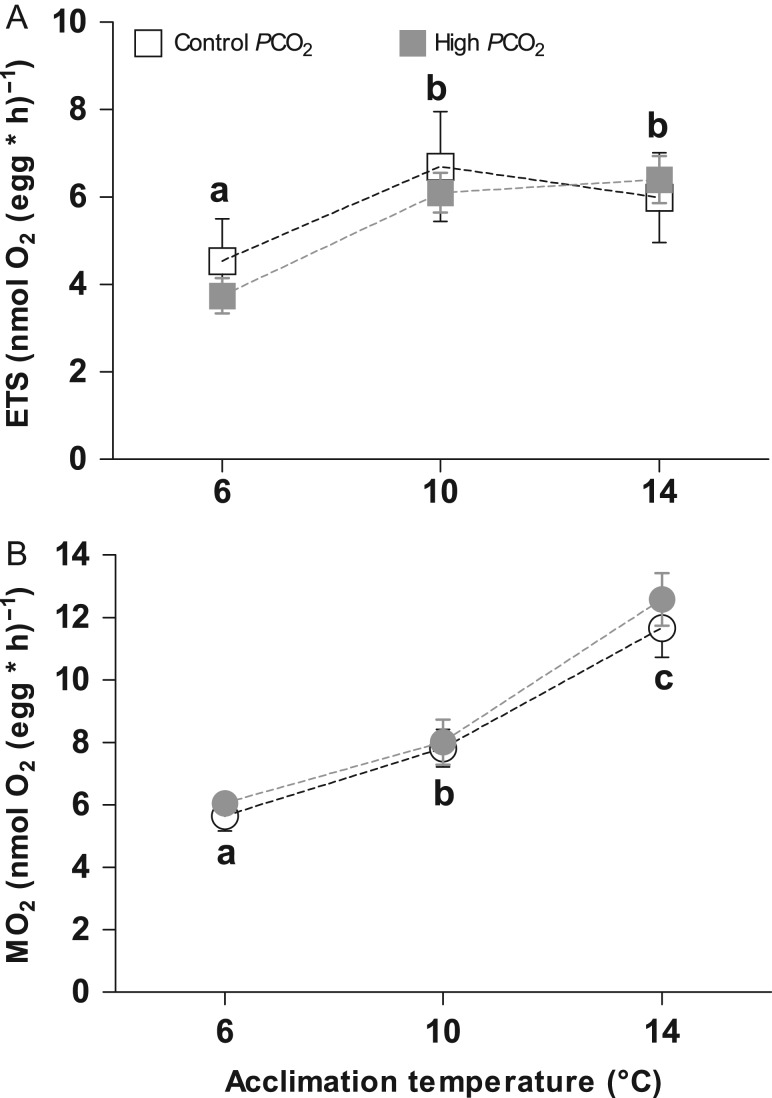
Respiration performance and mitochondrial capacity of Atlantic herring (*Clupea harengus*) embryos at 50% eye pigmentation stage. Values are reported as mean ± SEM. Panel **A**: Electron Transport System (ETS) capacity. Open squares: control *P*CO_2_ (400 μatm), solid squares: high *P*CO_2_ (1100 μatm). Panel **B**: Whole-embryo respiration. Open circles: control *P*CO_2_ (400 μatm), solid circles: high *P*CO_2_ (1100 μatm). Different letters within panels indicate significant differences (*P* < 0.05) between temperature treatments independent of the CO_2_ treatment.

The *in vivo* oxygen consumption rates (MO_2_) of Atlantic herring embryos were affected only by temperature (*F*= 173.87, *P* < 0.001, Fig. 1b). In general, MO_2_ increased with temperature in a non-linear fashion: expressed as *Q*_10_, the increase in MO_2_ between 6 and 10°C (Control *P*CO_2_: 2.30 ± 0.23; High PCO_2_: 2.00 ± 0.20) was lower than between 10 and 14°C (Control *P*CO_2_: 2.27 ± 0.14; High *P*CO_2_: 3.17 ± 0.27).

**Figure 2:**
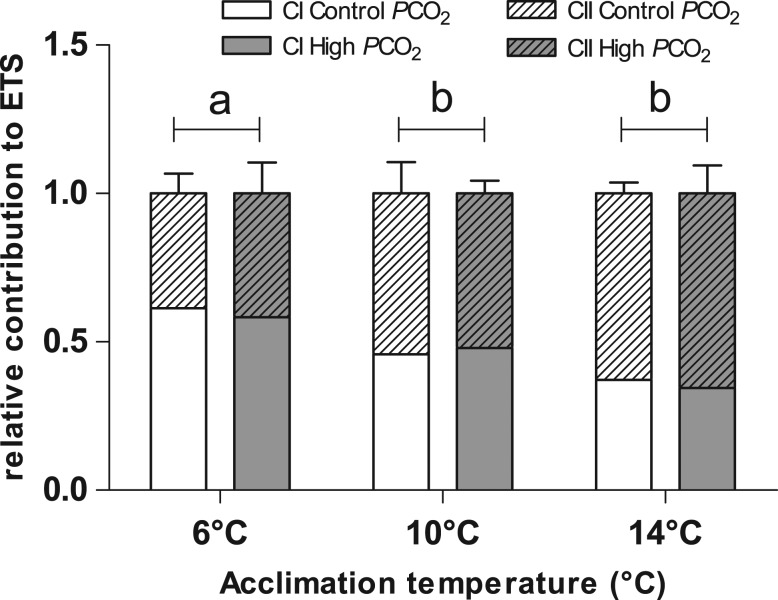
Contribution (%) of Complex I and Complex II to the electron transport system (ETS) in the embryos of Atlantic herring (*Clupea harengus*). Embryonic stage: 50% eye pigmentation. Values are reported as mean ± SEM. Open bars: Complex I, dashed bars: Complex II. Open bars: control *P*CO_2_ (400 μatm), solid bars: high *P*CO_2_ (1100 μatm). Different letters indicate statistical differences (*P* < 0.05) between temperature treatments independent of the CO_2_ treatment.

*In vitro*, the mitochondrial oxygen flux corresponding to the maximum capacity of the ETS was affected only by temperature (*F*= 5.61, *P* = 0.019, Fig. 1a) and increased between 6 and 10°C (*P* = 0.04), but, unlike whole-embryo MO_2_ (Fig. 1), reached a plateau between 10 and 14°C (*P* > 0.05). CI and CII contributed differently to the ETS according to temperature (*F* = 17.28, *P* < 0.001, Fig. 2). CI contribution declined with increasing temperature while CII contribution increased (Fig. 2). At 14°C, only 37% of the ETS capacity was contributed from CI, compared with 62% at 6°C (Fig. 2).

### Viable hatch and length at hatch

**Figure 3:**
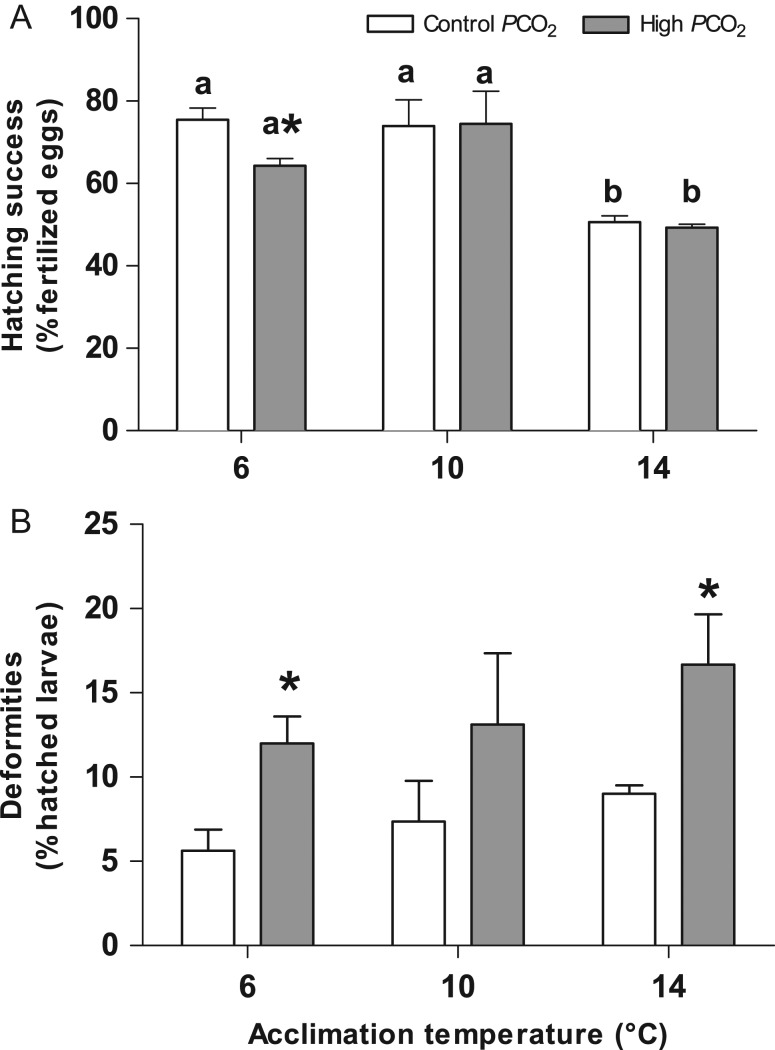
Viable hatch of Atlantic herring (*Clupea harengus*). Values are reported as mean ± SEM. Panel **A**: hatching success as percentage of fertilized eggs that hatch. Open bars: control *P*CO_2_ (400 μatm), solid bars: high *P*CO_2_ (1100 μatm). Panel **B**: Larval malformations as percentage of hatched larvae. Open bars: control *P*CO_2_ (400 μatm), solid bars: high *P*CO_2_ (1100 μatm). Different letters indicate statistical differences (*P* < 0.05) between temperature treatments, * indicates significant differences (*P* < 0.05) between CO_2_ groups at the same temperature.

HS (Fig. 3) was significantly affected by temperature (*F* = 14.07, *P* = 0.001) with a reduction of hatched larvae at 14°C compared with the other acclimation groups (6–14°C: *P* = 0.005; 10–14°C: *P* = 0.001; Fig. 3a). Elevated *P*CO_2_ had no significant effects on HS but caused a significant reduction (*P* = 0.02681) of the HS in the group incubated at 6°C (64.26 ± 1.72%) compared to the group incubated at the same temperature but under control *P*CO_2_ (75.43 ± 2.85%).

The proportion of larvae hatching with severe morphological malformations was higher in the groups incubated under high *P*CO_2_ (*F* = 13.03, *P* = 0.004, Fig. 3b) with percentages almost doubled compared with the groups incubated under control *P*CO_2_ (tab. 1). Larval malformations were not significantly correlated with increasing temperatures (*F* = 1.67, *P* > 0.05) and there was no interactive effect between temperature and CO_2_.

**Figure 4:**
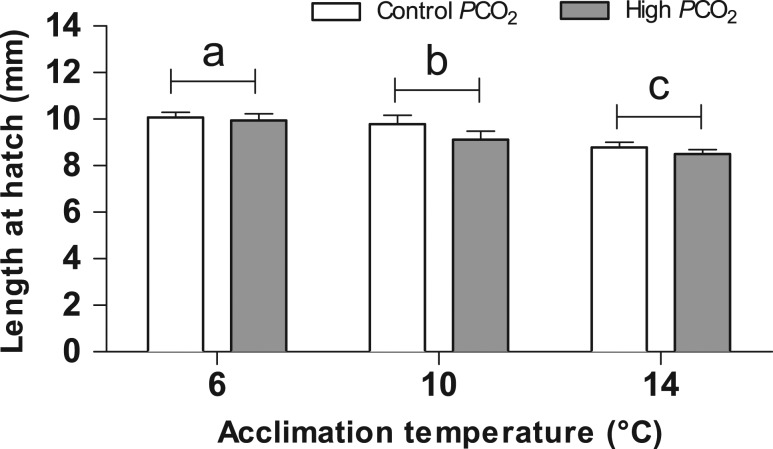
Length at hatch (mm) of Atlantic herring larvae (*Clupea harengus*). Values are expressed as mean ± SEM. Open bars: control *P*CO_2_ (400 μatm), solid bars: high *P*CO_2_ (1100 μatm). Different letters indicate significant differences (*P* < 0.05) between temperature treatments independent of the CO_2_ treatment.

SL at hatch (Fig. 4) was significantly affected by temperature (*F*= 43.12, *P* < 0.001) with a trend toward reduction with warming. SL was not affected by elevated *P*CO_2_ (*F* = 9.11, *P* > 0.05).

## Discussion

In this study, we analysed the development and mitochondrial function of Atlantic herring (*C. harengus*) embryos that were incubated to either current water conditions, or to conditions projected for the end of this century in waters surrounding the Scandinavian coast; one of the main spawning grounds of this species in the North Atlantic.

In general, we found that elevated temperature reduced HS and high *P*CO_2_ caused larval malformation. Mitochondrial function was not affected by elevated *P*CO_2_; however, temperature played a major role in shaping mitochondrial respiration, with subsequent effects on embryonic respiration and body length at hatching; which is in line with other studies on Atlantic herring (Geffen, 2002; Peck *et al.*, 2012).

The capacity of the ETS increased with temperature between 6°C and 10°C without a further increase at 14°C; however, the relative contribution of CI and CII to the ETS changed with temperature (for the entire range 6–14°C), with the contribution of CI being negatively correlated to temperature. In developing teleost fish, embryos mainly rely on carbohydrates during the initial phase of development, until blastula (Kamler, 2008), then catabolize amino acids from protein (benthophils) or free amino acids (FAA, pelagophils), together with lipids (Finn and Fyhn, 2010). In a study on Atlantic cod (*Gadus morhua*) eggs, Fyhn and Serigstad (1987) showed that the FAA content of the yolk was depleted by ~90% during spawning to hatching, but without a corresponding increase in the protein content of the developing embryo. Moreover, they found that alanine, serine, leucine, isoleucine, lysine, and valine (in that order) quantitatively dominated the amino acids pool, and accounted for ~75% of the decrease. Alanine, serine, leucine, isoleucine and lysine enter the TCA cycle at the citrate synthase step, via pyruvate (alanine and serine) or via acetyl-coA and acetoacetyl-coA (leucine, isoleucine and lysine); both of which are fed into CI and CII. Only valine and isoleucine enter the cycle via succinyl-coA and feed directly into CII. Taking this into account, the reduction of CI contribution to the ETS, in favour of CII, could indicate a shift in metabolic pathways from the preferred CI feeding amino acids (alanine, serine, leucine, isoleucine and lysine) to CII feeding amino acids (valine and isoleucine), as a result of increasing temperature. However, several studies have reported a reduced contribution of CI to the ETS with decreasing temperature in adult fish and embryos, with suggested causes being either a lack of substrates or a change in membrane fluidity (Hilton *et al.*, 2010; Iftikar *et al.*, 2015; Dahlke *et al.*, 2017). These two hypotheses are not contradictory, but complement (and even cause) each other.

A shift in ETS contribution from CI to CII results in a less efficient ATP production pathway, since each cycle of the TCA cycle theoretically produces ~7.5 ATP from CI, but only ~1.5 ATP from CII. The decreased ATP provision at higher temperatures would need compensation by an increase in embryonic respiration (MO_2_) as seen at 14°C in this study. In addition to the shift in the contribution of the individual complexes to ETS, increasing temperature may also cause a rise in mitochondrial uncoupling, increasing oxygen demand to compensate for the increased proton leak (Weinstein and Somero, 1998; Hardewig *et al.*, 1999). Therefore, an animal’s respiratory rate (MO_2_) may increase in order to partially compensate for these constraints. However, a limit may be reached where the animal is no longer able to provide oxygen to mitochondria or aerobically produce enough ATP; which may lead to constraints on performance, the onset of anaerobic metabolism and eventually death (Hardewig *et al.*, 1999; Pörtner, 2002).

In this study, we identified several negative effects of decreased ATP production efficiency at a higher incubation temperature (14°C). There was a decreased HS at this elevated temperature and the larvae that hatched at 14°C were smaller than the larvae from other incubation temperatures, indicating that less energy was available for development. Therefore, high temperature (14°) may have limited mitochondrial function, which is mirrored at the whole-organism level, by the decreased length and HS. This provides a link between thermal sensitivity of energy metabolism and the effects of warming at the whole-organism level.

Elevated *P*CO_2_ caused a significant increase in larval deformities. This is similar to the findings of Frommel *et al.* (2014), which showed that elevated *P*CO_2_ caused significant organ damage and reduced growth in the larvae of Atlantic herring. However, in another study on Atlantic herring embryos, (Franke and Clemmesen, 2011) found no significant effect of elevated *P*CO_2_ (levels up to 4635 μatm) on egg mortality or the occurrence of embryonic malformations. These contrasting findings could be partially explained by the different origins of the herring populations. The herring used in this study and the study by Frommel *et al.* (2014) came from the Scandinavian coast, while the herring used in the study by Franke and Clemmesen came from the Kiel Fjord in the Baltic Sea, where *P*CO_2_ levels are above 2300 μatm due to upwelling events (Thomsen *et al.*, 2010). Atlantic herring display high plasticity in physiological tolerance (Geffen, 2009; Peck *et al.*, 2012), allowing different populations to spawn in different seasons and live in a broad range of temperatures and salinities (Geffen, 2009). Herring lay adhesive benthic eggs (Nash *et al.*, 2009; Schmidt *et al.*, 2009) and therefore encounter potentially challenging hydrographic conditions during egg development, since bottom waters are often depleted of oxygen and enriched in CO_2_, relative to surface waters. These results contribute to the growing evidence of differences in the sensitivity towards Ocean Acidification and Warming between herring populations (Franke and Clemmesen, 2011; Sswat *et al.*, 2018) and compared to pelagic spawners such as Atlantic cod, flounder and many tropical reef species (Chambers *et al.*, 2014; Munday *et al.*, 2016; Dahlke *et al.*, 2017).

## Conclusions

Our study assessed the effects of combined Ocean Acidification and Warming on developing eggs of Atlantic herring. By studying such effects at both the cellular level (e.g. mitochondrial functioning) and the organism level (e.g. body size at hatching), this study provides a link between the thermal sensitivity of an individual’s energetic metabolism with the fitness of the individual as a whole.

Elevated temperature significantly affected mitochondrial function by shifting the relative ETS contribution from CI to CII. This may decrease ATP production, which could lead to a mismatch between the energy produced by the mitochondria and the energy requested by the organism for maintaining metabolism; which in turn could reduce the energy allocation to development indicated by reduced length at hatch.

Elevated *P*CO_2_ did not affect HS; however, it did increase the occurrence of malformed larvae. This suggests that exposure to near future acidification levels may cause sub-lethal cellular damage that may not be reflected in vitality and survival rates. These sub-lethal effects of ocean acidification may present the largest risk to individuals and populations (Briffa *et al.*, 2012). For example, smaller larval size at hatch may increase the risk of predation and reduce foraging ability (Miller *et al.*, 1988).

Furthermore, herring populations experience high fishing mortality in addition to other environmental stressors such as pollution and hypoxia. Therefore, potential effects of ocean acidification and warming must be added to the list of anthropogenic perturbations leading to increased mortality in fish early life stages.

## Supplementary Material

Supplementary Data

## Data Availability

The datasets containing the physiological and morphological parameters measured in this study and the data regarding the incubation physico-chemical parameters are available from the Open Access library PANGAEA (www.pangaea.de; https://doi.pangaea.de/10.1594/PANGAEA.884123 and https://doi.pangaea.de/10.1594/PANGAEA.884124).
