# Supplementary material for: Impact of Ocean Acidification and Warming on the bioenergetics of developing eggs of Atlantic herring Clupea harengus
Source: Conserv Physiol. 2018 Sep 18;6(1):coy050. doi: 10.1093/conphys/coy050 (PMC6142905; doi:10.1093/conphys/coy050)
Supplement: Supplementary Data [file coy050_leo_et_al_supplementary_material.docx]

**Supplementary material**

Impact of Ocean Acidification and Warming on the bioenergetics of developing eggs of Atlantic herring *Clupea harengus*.

Elettra Leo1,2, Flemming T. Dahlke1,2, Daniela Storch1, Hans-O. Pörtner1,2, Felix C. Mark1,*

1 Alfred Wegener Institute Helmholtz Centre for Polar and Marine Research, Integrative Ecophysiology, Am Handelshafen 12, D-27570 Bremerhaven, Germany

2 University of Bremen, Fachbereich 2, NW 2 / Leobener Strasse, D-28359 Bremen, Germany

*Corresponding author: Alfred Wegener Institute Helmholtz Centre for Polar and Marine Research, Integrative Ecophysiology, Am Handelshafen 12, D-27570 Bremerhaven, Germany.

Email: Felix.Christopher.Mark@awi.de

**Figure S1.** (A) Incubator and (B) Incubation setup. (A) Herring eggs were incubated in closed incubators with a water volume of 1 L. (B). The incubators were placed into temperature-controlled water baths (1) set to 6, 10 and 14 °C. Sterilized seawater used for daily water exchange was adjusted to the respective temperature and *P*CO_2_ within submerged reservoir tanks (2a, b). Elevated CO_2_ concentrations (*P*CO_2_: 1100 µatm, pH: 7.7) were administered via infusion of pure CO_2_ (2b) using a computer-controlled feedback system (3). Both reservoir tanks (control, high- CO_2_, 60 L) were bubbled with pressured air to ensure high oxygen saturation (>95%) as well as homogeneous CO_2_ concentrations and fast equilibration of seawater pH (within high- CO_2_ reservoirs). Tempered liquid (4) pumped through submerged tubing (5) was used to maintain constant water temperatures. Note that the drawings may not reflect realistic size ratios.


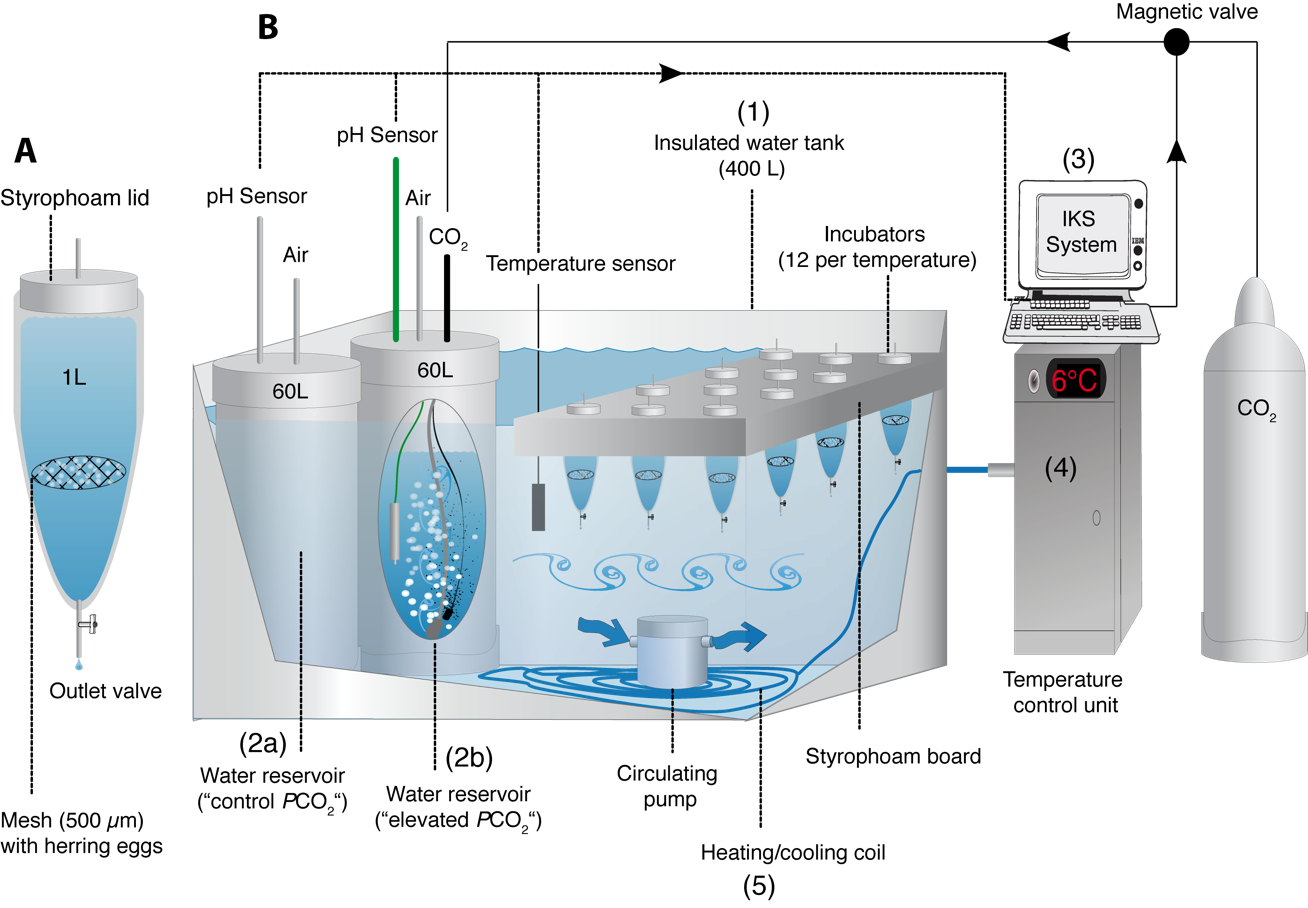


**Figure S2.** Respiration setup. Oxygen consumption rates of herring embryos were measured in closed, temperature-controlled respiration chambers. Both chambers were connected to a temperature control device (not shown) pumping tempered water through the double-walled glass bodies. Herring eggs were placed on a polyethylene mesh with a magnetic micro stirrer underneath to avoid oxygen stratification inside the respiration chamber (2 ml water volume). Note that the drawings may not reflect realistic size ratios. Pictures show herring eggs at the stage of 50% eye pigmentation. The pictures were taken during the staging procedure, not within the respiration chamber. Care was taken that the eggs within the respiration chamber were not attached to each other.


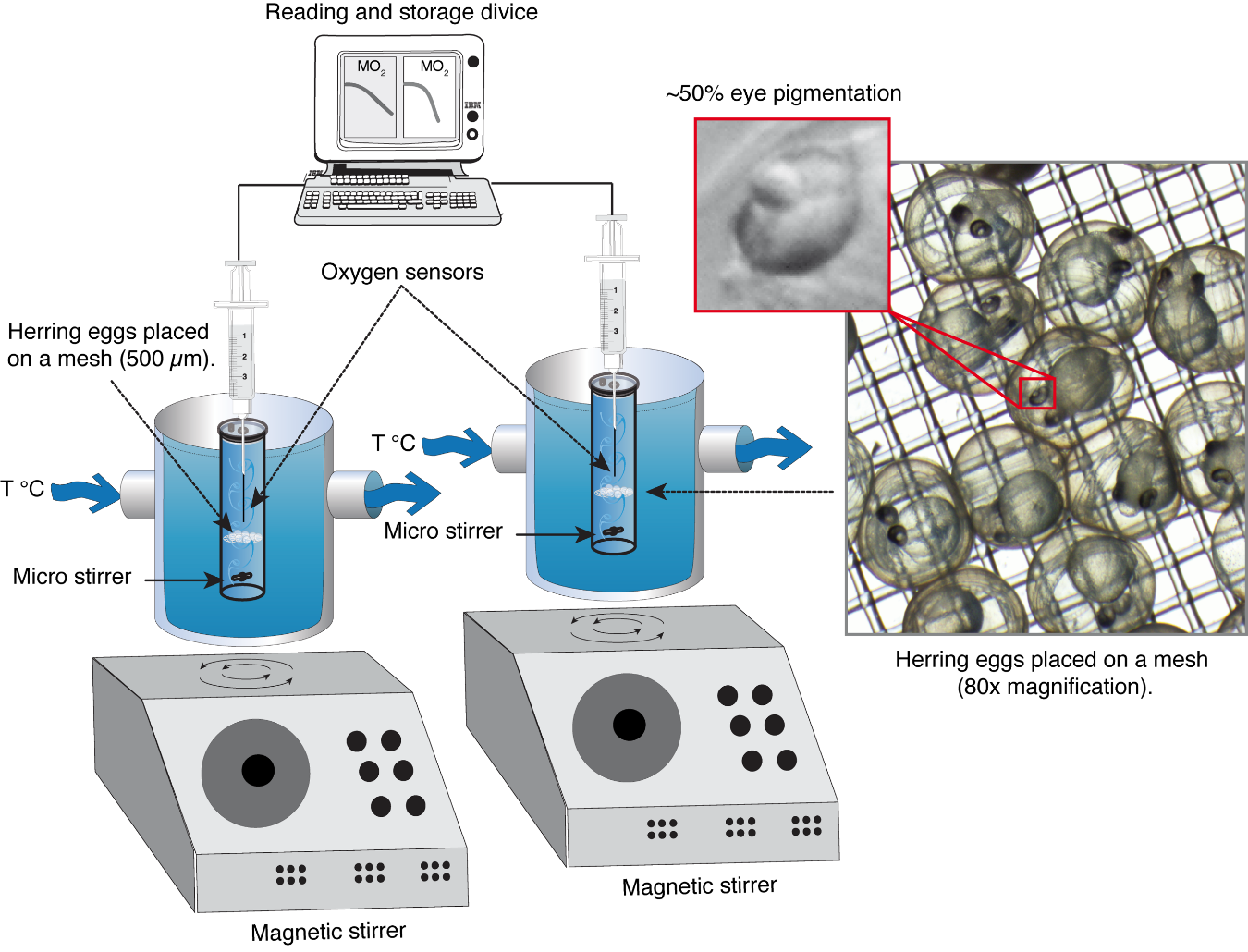


**Figure S3.** Temperature-dependent development times of herring eggs until 50% eye pigmentation (triangles) and peak hatch (circles). Development times did not vary between females and *P*CO_2_ treatments. The effect of temperature (*T*) on the rate of development (*D*) was described by power functions (pooled CO_2_ treatments, n = 6, p < 0.001, data are presented in Table S1). Symbols are means (± S.E.M., error bars fall within symbols).


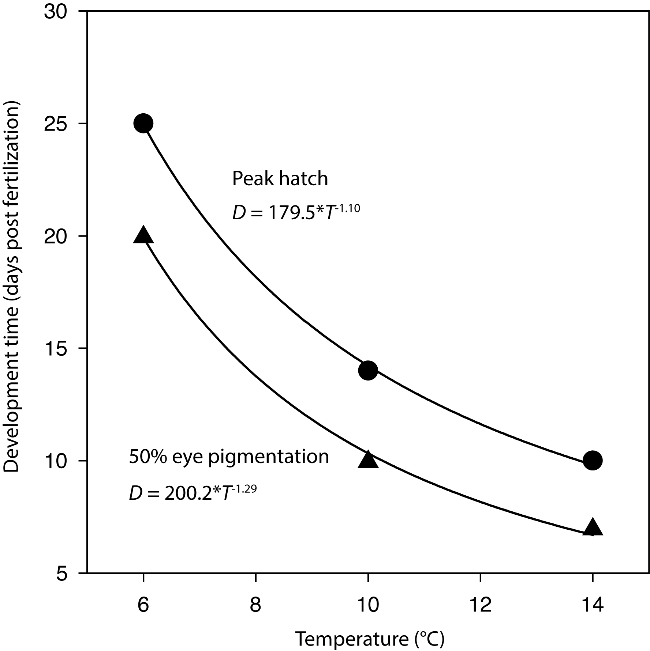


**Table S1.** Summary table for egg masses (wet weights) and development times of herring eggs that were produced by three different females and exposed to different temperature and *P*CO_2_ conditions. Egg masses were determined after respiration measurements (MO_2_) at the stage of 50% eye pigmentation. Staging was done by visual inspection during daily water exchange. Peak hatch was defined as the day post fertilization (dpf) when more than 50% of the larvae had hatched. Egg masses and development times did not differ between *P*CO_2_ treatments (ANOVA, p > 0.5). The effect of temperature on the time until 50% eye pigmentation and peak hatch was described by power functions (Figure S3).

| Female | Temperature (°C) | *P*CO2 (µatm) | Egg mass (mg) | 50% eye pigmentation (dpf) | Peak hatch (dpf) |
| --- | --- | --- | --- | --- | --- |
|  |  |  |  |  |  |
| 1 | 6 | 400 | 2.49 | 20 | 25 |
| 2 | 6 | 400 | 2.79 | 20 | 25 |
| 3 | 6 | 400 | 1.79 | 20 | 25 |
| 1 | 10 | 400 | 2.65 | 10 | 14 |
| 2 | 10 | 400 | 2.82 | 10 | 14 |
| 3 | 10 | 400 | 1.72 | 10 | 14 |
| 1 | 14 | 400 | 2.61 | 6 | 10 |
| 2 | 14 | 400 | 2.71 | 6 | 10 |
| 3 | 14 | 400 | 1.83 | 6 | 10 |
| 1 | 6 | 1100 | 2.55 | 20 | 25 |
| 2 | 6 | 1100 | 2.39 | 20 | 25 |
| 3 | 6 | 1100 | 1.68 | 20 | 25 |
| 1 | 10 | 1100 | 2.36 | 10 | 14 |
| 2 | 10 | 1100 | 2.39 | 10 | 14 |
| 3 | 10 | 1100 | 1.70 | 10 | 14 |
| 1 | 14 | 1100 | 2.79 | 6 | 10 |
| 2 | 14 | 1100 | 2.76 | 6 | 10 |
| 3 | 14 | 1100 | 1.49 | 6 | 10 |
